# Etherization

**Published:** 1866-09

**Authors:** 


Etherization.—M. Velpeau has communicated to the Academy some remarks of M. Petrequin, of Lyons, about etherization. Ether, employed by Dr. Jackson in 1846, was replaced by chloroform in 1849. Chloroform, as a chemical body, was discovered by MM. Soubeiran and Pelouze; and its physiological action was tried for the first time by M. Flourens. But, if chloroform acts more rapidly than ether as an anæsthetic, it is also more destructive in its effects; and consequently surgeons in the south of France never employ it. M. Buisson, in a work which received a recompense from the Academy some years ago, wrote against chloroform—an agent "wonderful and terrible," as Flourens said of it. M. Petrequin now tells us that for fifteen years the surgeons of Lyons have employed ether only, and that they have never lost a patient. M. Velpeau hereon observed, that fatal accidents had been much more frequent abroad, and especially in England, than in France, and in the departments of France than in Paris. Much, therefore, depends upon the care with which chloroform is used. He had himself put thousand of patients under chloroform, and had never met with the slightest accident.—[British Medical Journal, Jan. 6, 1866.

